# Methods for Developing a Process Design Space Using Retrospective Data

**DOI:** 10.3390/pharmaceutics15112629

**Published:** 2023-11-16

**Authors:** Miquel Romero-Obon, Pilar Pérez-Lozano, Khadija Rouaz-El-Hajoui, Marc Suñé-Pou, Anna Nardi-Ricart, Josep M. Suñé-Negre, Encarna García-Montoya

**Affiliations:** 1 Laboratorios ALMIRALL, Ctra. de Martorell, 41-61, 08740 Sant Andreu de la Barca, Spain; miquel.romero@ub.edu; 2 Department of Pharmacy and Pharmaceutical Technology and Physical Chemistry, Faculty of Pharmacy and Food Sciences, University of Barcelona, Av. Joan XXIII, 27-31, 08028 Barcelona, Spain; khadijarouaz@ub.edu (K.R.-E.-H.); marcsune@ub.edu (M.S.-P.); annanardi@ub.edu (A.N.-R.); jmsune@ub.edu (J.M.S.-N.); encarnagarcia@ub.edu (E.G.-M.); 3 Pharmacotherapy, Pharmacogenetics and Pharmaceutical Technology Research Group, Bellvitge Biomedical Research Institute (IDIBELL), Av. Gran via de l’Hospitalet, 199-203, 08090 Barcelona, Spain

**Keywords:** design space, design of experiment, retrospective data, legacy product, collinearity, overfitting, principal component analysis, PCA, LASSO regression, wet granulation, kneading

## Abstract

Prospectively planned designs of experiments (DoEs) offer a valuable approach to preventing collinearity issues that can result in statistical confusion, leading to misinterpretation and reducing the predictability of statistical models. However, it is also possible to develop models using historical data, provided that certain guidelines are followed to enhance and ensure proper statistical modeling. This article presents a methodology for constructing a design space using process data, while avoiding the common pitfalls associated with retrospective data analysis. For this study, data from a real wet granulation process were collected to pragmatically illustrate all the concepts and methods developed in this article.

## 1. Introduction

Legacy products that predate the design space concept formulated by ICH Q8 [1] were not developed using the most up-to-date methods. This means that companies lack a set of equations describing how the laws of nature govern internal processes. Unfortunately, this outdated mindset not only applies to legacy products but also affects far too many products beyond what the pharmaceutical industry would prefer. Researchers in the pharmaceutical industry recognize the value of industrial data and, as a result, have published studies on various pharmaceutical forms using traditional statistical tools [2,3,4,5,6,7] and, more recently, employing AI [8,9,10].

In these circumstances, product knowledge and process understanding rely more on manufacturing experience than on design, while regulatory constraints further inhibit continuous improvement, as anticipated by ICH Q10 [11].

Classical statistical methods, such as multiple linear regression (MLR), require linearity, homoscedasticity, independence, and normality of residuals. Once these prerequisites are confirmed, the resulting models should be employed when both explainability and predictability are deemed sufficiently robust, and the parsimony principle is upheld to prevent overparameterization. However, this widely used approach does not take into consideration whether data were collected prospectively or retrospectively. This consideration is crucial to establish the validity of the discovered statistical model or to discard it due to the potential hidden effects of multicollinearity, which can lead to unstable coefficients in the regression equation (resulting in weak characterization of the design space) and statistical confusion (leading to incorrect inferences from data that seemingly suggest a relationship between regressors and responses when it is actually weak or nonexistent).

This article contributes to the identification of common methodological errors and provides solutions that enable the use of retrospective data for process design space construction. The proposed methodology can speed up the QbD implementation in the pharmaceutical industry and significantly facilitates process understanding. In addition, using industrial processes and automatically collected real-time data provides novelty to the QbD science field.

## 2. Background

### 2.1. Understanding Wet Granulation

Before delving into statistics, it is essential to have a comprehensive understanding of the process under consideration in terms of galenic, physicochemical, and thermodynamic aspects.

Powder mixtures may exhibit low rheology and compressibility, leading to poor or even non-processability in tablet manufacturing. Both characteristics can be estimated using the Carr index and Hausner ratio, which are calculated based on the same two parameters: bulk density and tapped density. Rheology and/or compressibility can be improved by physically modifying the powder to create an aggregated mixture, where new and larger particles are formed from a blend of excipients and active principal ingredient (API). These aggregates are formed with the help of a binding agent, typically containing water (although some organic solvents can also be used). After the powder is premixed, the binding solution is added to it under controlled conditions of flow rate and time. Kneading begins by stirring the wet powder in a granulator, transforming individual particles into aggregates. This wet mixture will undergo three distinct phases during this stage: densification, transition, and dilatation.

Densification occurs when the dry powder becomes wet, leading to a reduction in its volume. During this phase, the power consumption by the granulator impeller exhibits a slight increase. Upon reaching the transition phase, the wet powder consists of agglomerated particles with a significantly higher power consumption. If the process exceeds the saturation curve, it may transform into a paste with undesirable characteristics, deviating from the granulation objectives. Generally, this paste is not useful, and the process will typically result in the rejection of the processed batch.

In summary, the optimal point to conclude granulation falls within the transition phase and should not be surpassed. Impeller power, as an outcome of the process (rather than a predefined critical process parameter) is used to make decisions about the process endpoint.

**Figure 1:**
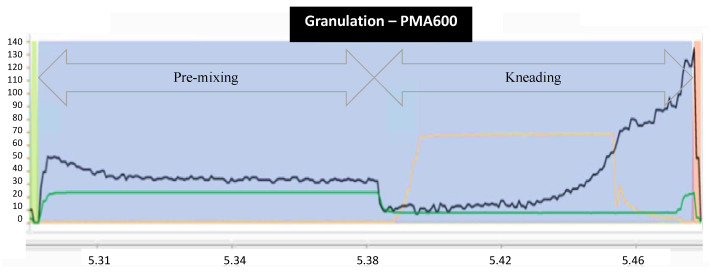
Real-time monitoring of a granulation process using GEA PMA600 (GEA, Düsseldorf, Germany) equipment (X-axis for time in minutes and Y-axis for the impeller speed (green line), impeller power consumption (blue line), and binding liquid addition (yellow line).

The granulation process is illustrated in Figure 1 in terms of critical process parameters, with impeller speed (green line), impeller power consumption (black line), and binding liquid addition (orange line) against process time on the X-axis. Double arrows indicate the premixing and kneading steps.

During the premixing step, the impeller operates at a constant speed of 22 rpm, with power consumption remaining consistently around 40 KWh. Once the binding solution is added, the kneading step occurs at a constant impeller speed of 10 rpm. The wet product exhibits increased resistance to kneading, with the impeller’s power consumption peaking at 130 KWh in the impeller power consumption. This represents the optimal point at which the process should cease; otherwise, it will transform into an unusable paste. If the process stops prematurely, partial agglomeration occurs, resulting in a poorly compressible intermediate product.

Due to the inherent variability in particle size, weight tolerances, adjustments related to potency/assay, acceptable measurement errors of process sensors, and human perception, this process will exhibit a profile-like fingerprint rather than an exact batch-to-batch exact match.

### 2.2. Understanding Statistics

To identify an appropriate approach to creating statistical models that can effectively minimize overfitting, account for influential factors beforehand, and address collinearity issues, it is crucial to comprehend their origins, impacts, and methods for measuring them. 

#### 2.2.1. Overfitting

Overfitting is an issue that arises in modeling when the model closely matches a specific dataset but may struggle to make reliable predictions for future observations. In overfitted statistical models, there are more parameters (predictors) included than really needed. Although overfitted models can be used for descriptive purposes, they will not properly work for predictive ones.

While overfitted models often do not exhibit bias in their parameter estimations, they tend to have unnecessarily large, estimated sampling variances, resulting in poor precision of the estimations compared to what could be achieved with a more parsimonious model. This can lead to the identification of false treatment effects and the inclusion of irrelevant variables in overfitted models.

#### 2.2.2. Influent Individuals

An a priori influential value is an outlier with respect the predictor population. These values, also known as high-leverage points, have no neighboring points and have the potential to significantly impact the parameter estimates. While influential points typically exhibit high leverage, it is important to note that not all high-leverage points are necessarily influential. The identification of high-leverage points can be accomplished using the formula shown in Equation (1) and applying the criteria *h_i_* ≥ 2*p*/*n* as a threshold, where *p* represents the number of parameters, and *n* represents the number of individuals. Equation (1) shows the leverage calculation.

(1) $$h_{i}=\frac{1}{n}+\frac{{\left(X_{i}-\bar{X}\right)}^{2}}{\sum_{j=1}^{n}{\left(X_{j}-\bar{X}\right)}^{2}}$$

where *h_i_* is the leverage value for point *i*, *n* is the number of individuals, $\bar{X}$ is the average of the *X* values, *X_i_* is the value of *X* for the evaluated point *i*, and *X_j_* represents each value of *X* from 1 to *n.*

This effect can be assessed by recalculating the statistical model with and without the suspicious point. This is the purpose of Cook’s distance parameter, which can be calculated as shown in Equation (2). Cook’s distance is also referred to as an a posteriori influence since it measures the effect on the model estimation rather than assessing the individual point in relation to the predictor population. Equation (2) shows Cook’s distance formula.

(2) $$D_{i}=\frac{\sum_{j=1}^{n}{\left({\hat{Y}}_{j}-{\hat{Y}}_{j\left(i\right)}\right)}^{2}}{\left(p+1\right)\;{\hat{\sigma }}^{2}}$$

where *D_i_* is Cook’s distance, *p* is the number of parameters in the model, ${\hat{\sigma }}^{2}$ is the estimated variance, and $\left({\hat{Y}}_{j}-{\hat{Y}}_{j\left(i\right)}\right)$ are the differences between predictions made with all observations in the analysis and predictions made leaving out the observation in question.

Since this value (*D_i_*) tends to approach 1 for large *n*, a simple operational guideline of using *D_i_* > 1 as a threshold has been suggested [12].

#### 2.2.3. Requirements of Linear Models

Linear Models are based on four fundamental requirements that must be verified before drawing conclusions about the model’s quality: linearity, normality, homoscedasticity, and independence.

**Figure 2:**
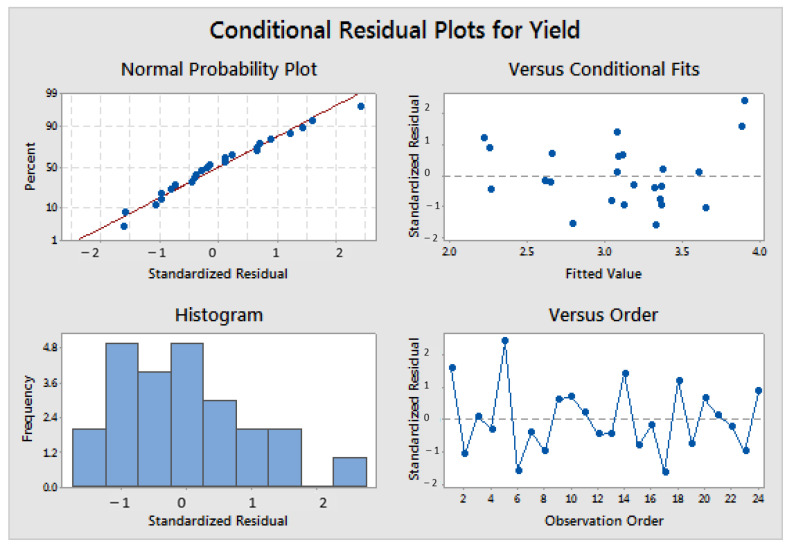
Example of graphical assessment of normality, homoscedasticity, and independence of residuals (made using the statistical software Minitab v21).

Often, these requirements are overlooked when the obtained results align with prior expectations. To avoid making this common mistake, researchers must assess the preconditions of linear models. Graphical methods, as shown in Figure 2, are usually sufficient and widely accepted. The upper and bottom left plots in Figure 2 pertain to normality, the upper right plot illustrates homoscedasticity or equal variance against fitted values, and the bottom right graph depicts independence versus time.

When the linearity requisite is not met, consideration should be given to second-term parameters, such as the square of the variable and/or second-order interactions). For more complex non-linear functions, such as polynomic regression, Gompertz or Logistic Growth, among other models, can be considered (not in the scope of this article).

If homoscedasticity is not achieved, a mathematical transformation of the response variable can be applied (e.g., Johnson or Box–Cox transformation). Afterward, homoscedasticity should be re-evaluated, as this method does not guarantee a 100% success rate in all cases. If the data exhibit heteroscedasticity that cannot be rectified as mentioned, it is essential to maintain a conservative interpretation of the model.

Regarding the normality of residuals, if it does not hold, there are two options available: applying a mathematical transformation (which can address both heteroscedasticity and the lack of normality simultaneously) or utilizing a non-parametric statistical model (e.g., Kruskal–Wallis).

**Figure 3:**
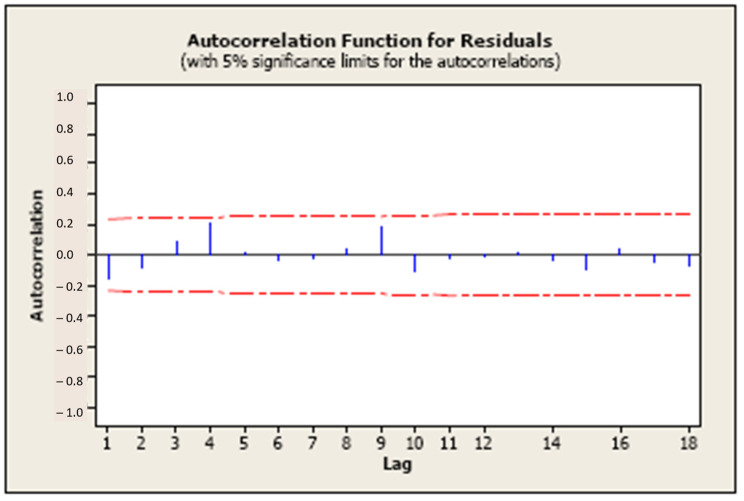
Autocorrelation function plot to check independence over time. Bars exceeding the 5% significance limits (red line) would show dependence on previous result/s (made using the statistical software Minitab v21).

Lastly, independence between residuals and time must be assessed to ensure there are no autoregressive effects (time-dependent response that could lead to spurious relationships between predictor/s and response/s). Independence can be verified by examining an autocorrelation function plot, where the bars should not exceed the limits, as shown in Figure 3.

#### 2.2.4. Sources and Effects of Collinearity

Collinearity arises from correlations between predictive variables, indicating significant correlations between critical process parameters (CPP) and critical material attributes (CMA). When working with larger datasets containing a high number of variables, spurious correlations can emerge. The larger the number of variables, the more likely this is to occur.

Models with highly correlated predictive variables will yield unstable regression coefficients. In simpler terms, inadequate equations will be obtained to describe the design space.

To identify and measure collinearity, both numerical and graphical methods can be employed. When correlation exists between two variables, the Pearson correlation coefficient (*r*) and correlation matrix can be used for quantitative and qualitative purposes, respectively (see Equation (3)). Equation (3) shows the Pearson correlation coefficient. *S_XY_* represents the covariance between *X* and *Y*.

(3) $$r=\frac{S_{XY}}{S_{X\;}S_{Y\;}}$$

*S_X_* is the standard deviation of *X. S_Y_* is the standard deviation of *Y*.

When dealing with more than two predictive variables, the variance inflation factor (VIF) becomes a useful measure for detecting multicollinearity. VIF is calculated as the ratio of the variance of a coefficient in a model with multiple predictors to the variance of that coefficient in a model with only one predictor. The acceptance criteria are as follows: when VIF < 1, the variables are not correlated; if 1 < VIF ≤ 5, moderate multicollinearity can be assumed; if VIF > 5, then variables are highly correlated.

#### 2.2.5. Compensating for Collinearity

When using MLR for explanatory or modeling purposes, collinearity can result in unstable regression coefficients and inaccurate equations. Since identifying collinearity is crucial for rejecting inappropriate statistical models, the first strategy is to discard any model displaying high levels of VIF. While this serves as a protective measure, it is not a complete solution. So, what should be done when collinearity is identified?

This article discusses two different methods for compensating for collinearity:

- Principal component analysis (PCA) before the regression study.
- Least absolute shrinkage and selection operator (LASSO) regression.

### 2.3. Principal Component Regression

PCA is a statistical technique designed to reduce dimensionality. When dealing with a larger number of predictors, PCA offers the opportunity to work with fewer variables than the original set [13]. This approach is based on finding a new coordinate system with orthogonality properties, meaning that all variables are independent of each other. The new coordinate system is calculated as a linear combination of the original variables, ensuring orthogonality. It can be used to conduct an MLR study, with the advantage of having fewer variables and no collinearity.

However, this method has its drawbacks, including the potential loss of variance explainability due to improper dimensionality reduction (selecting an incorrect number of predictors) and the increased complexity of interpreting the new variables, which are linear combinations of the original variables rather than the CPPs and CMAs.

### 2.4. LASSO Regression

LASSO [14] is a regression analysis method, also known as L1 regularization, that combines variable selection and regularization to improve the prediction accuracy and interpretability of the resulting statistical model. LASSO achieves this by applying a penalty factor to each predictor. This penalty factor proportionally reduces the coefficient when collinearity is detected, resulting in the effective tuning of model parameters, even in the presence of correlations between predictors [15]. In some cases, this reduction can entirely eliminate a variable (coefficient = 0), indicating that the corresponding process parameter or material attribute should not be included in the equation (as it is not a true CPP or CMA).

This method is valuable for objectively identifying CPPs and CMAs, reducing subjectivity in previous FMEA studies. This objectivity aligns with the requirements outlined in ICH Q9 (R1), Chapter 5.3, “Managing and minimizing subjectivity” [16], which was moved to step 5 by the US-FDA in May 2023 and by the EMA for Europe in July 2023.

## 3. Methods and Materials

A retrospective dataset was utilized to illustrate the concepts mentioned above, which help improve statistical models while avoiding common errors when dealing with historical results.

The dataset included seven material attributes (d10, d50, and d90 to characterize the granulometric profile of the main excipient and API, as well as the moisture content of the main excipient) and six process parameters (premixing impeller speed, premixing time, binder addition flow rate, impeller speed during kneading, and kneading time). Impeller power consumption during kneading was also automatically recorded at the same frequency as the CPPs (1 result/second).

All process parameters were captured in real time using installed sensors in a granulator GEA PMA600 and processed using Ignition^®^ 8.1 (Inductive Automation, Folsom, CA, USA) and Minitab^®^ 21 (Minitab, LLC, State College, PA, USA) software. LASSO regression analysis was conducted using specific Python (Python Sotware Fundation, Troisdorf, Germany) scripts, as most available statistical software lacks this feature.

The study timeframe ranged from 7 August to 4 September 2023, covering the manufacturing period of 30 batches of the same product (name not given for confidentiality reasons). For each batch, the following parameters were captured by equipment sensors:

- Impeller speed (rpm).
- Impeller power consumption (KWh).
- Binder solution flow (g/min).
- Premixing time (s).
- Kneading time (s).

Also, for each batch, the listed material attributes were collected:

- API d10: particle size that characterizes the 10th percentile of the API granulometric distribution.
- API d50: average of the API particle size.
- API d90: particle size that characterizes the 90th percentile of the API granulometric distribution.
- Lactose d10: particle size that characterizes the 10th percentile of the lactose granulometric distribution.
- Lactose d50: average of the lactose particle size.
- Lactose d90: particle size that characterizes the 90th percentile of the lactose granulometric distribution.
- Lactose moisture: water content in lactose expressed as a percentage of the total mass.

The following scripts were specifically written in Python for this study:

- LASSO regression.
- False Discovery Proportion vs. lambda parameter in LASSO regression [17].

The Python code is included in Supplementary Materials File S1 for the mentioned algorithms.

## 4. Proposed Methodology

**Figure 4:**
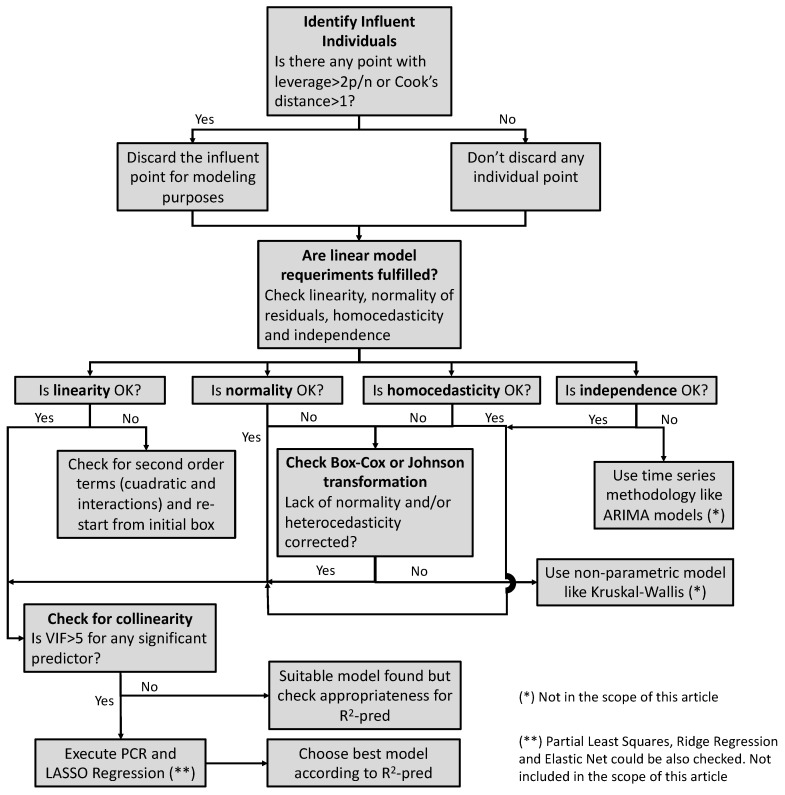
Decision tree to identify statistical pitfalls and paths to minimize their impact.

The following decision tree (Figure 4) depicts the procedure to follow to consider each of the potential statistical artifacts and how to minimize them to obtain suitable mathematical models with improved prediction features.

## 5. Results and Discussion

The same dataset was statistically analyzed using the following methods.

- Direct statistical processing with MLR.
- Principal component regression extracted from the original data.
- LASSO regression.

In all cases, the models were constructed using 75% of the data, while the remaining 25% were reserved for model validation. The results and conclusions from each statistical method are presented and discussed in the subsequent chapters.

### 5.1. Processing via Conventional MLR

**Figure 5:**
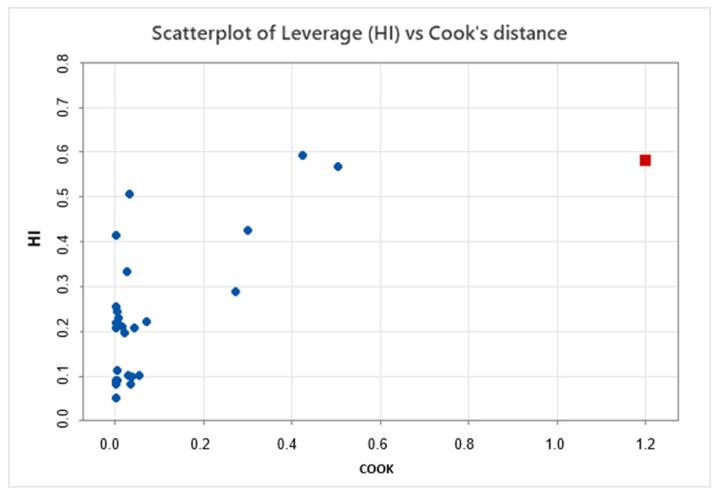
Leverage (HI) vs. Cook’s distance to identify influential points. The red point shows strong influence as it overcomes the conventional threshold (Cook’s distance > 1.0 for this individual), meaning that this point has a strong effect and should be avoided for modeling purposes.

MLR, also known as ordinary least squares, was applied to the entire dataset. Influential values were identified by calculating the leverage and Cook’s distance. The model estimation with and without the influential values displayed significant differences, and R^2^ also experienced a notable change. Both effects indicate the instability of model coefficients when influential values are present (refer to Figure 5).

The significantly different coefficients observed in both equations (with and without the influential point) highlight the instability and weakness of the model generated via the MLR method for this dataset.

Upon removing the identified influential values, VIF calculation reveals strong collinearity (as shown in Table 1). Once again, the MLR model proves unreliable; although its R^2^ value appears satisfactory, the resulting equation is only suitable for describing the current dataset, lacking the ability to predict new outcomes. Refer to the bottom of Table 1 to see how R^2^ decreases when moving to prediction and 10-fold cross-validation. The results indicate that the model can solely serve a descriptive purpose, as its predictive capability is compromised, evident in the significantly lower R^2^ (pred) compared to R^2^.

If the variable with the highest VIF (lactose moisture and related second-term parameters) is removed from the model, the resulting equation becomes unreliable, with very low predictability (R^2^-pred = 48%). For obvious reasons, it should not be used to describe the process design space.

### 5.2. MLR on Principal Components

**Figure 6:**
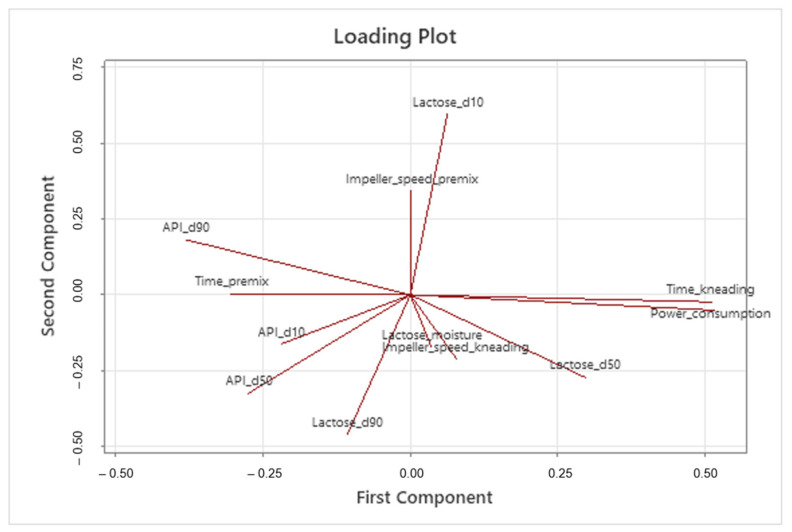
Loading plot showing variables’ relationships. Kneading time is strongly correlated with power consumption.

After calculating the principal components, all variables are redefined using the new coordinate system. Additionally, the CPPs, CMAs, and CQAs are visually presented in a loading plot, aiding in their identification and the understanding of their relationships (refer to Figure 6).

Variables demonstrating orthogonality (e.g., API_d90 and Lactose_d10) can be considered independent, while those pointing in the same direction should be understood as correlated. They can be positively correlated if they share the same direction and sense (e.g., time_kneading and power_consumption) or negatively correlated if they share the same direction but not the same sense (e.g., Lactose_d10 and Lactose_d90). Scientists should exercise caution when interpreting the loading plot because correlation does not imply causality.

**Figure 7:**
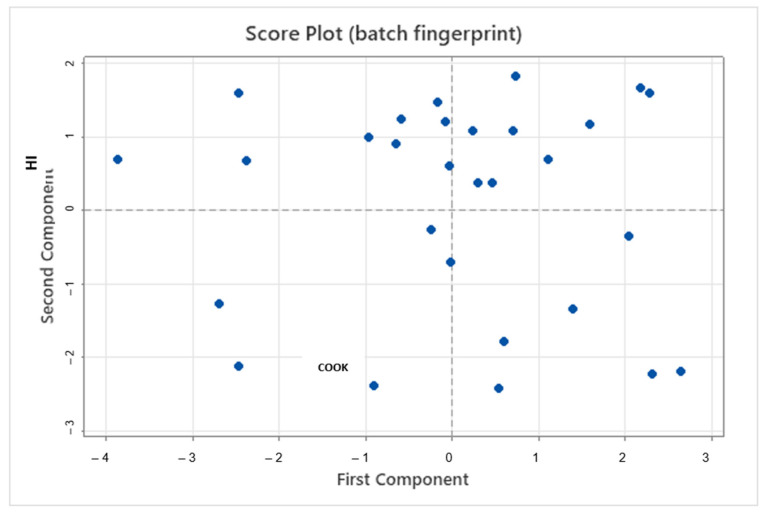
Score plot (batch fingerprint) of first and second principal components.

Individual batches are also represented using the first and second principal components, simplifying the multidimensional representation, commonly referred to as a batch fingerprint. Clusters indicate batch similarities, while separation between points indicates different profiles (see Figure 7).

**Figure 8:**
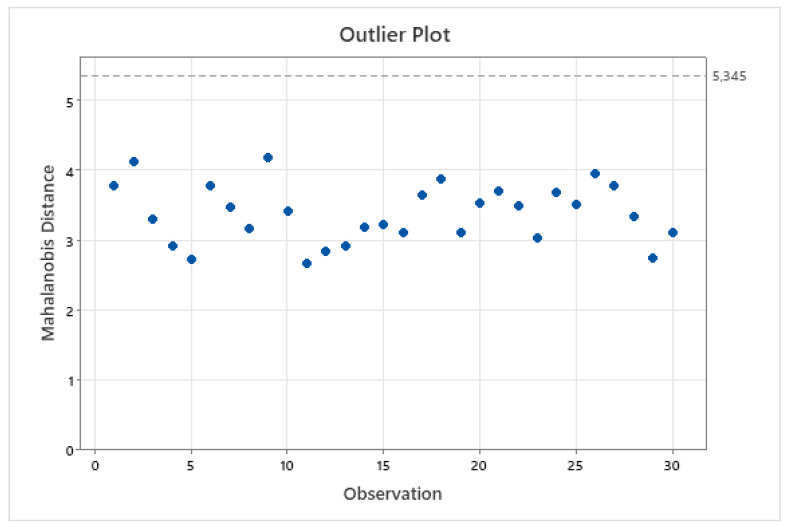
Mahalanobis distance used to identify outliers. No points fall outside the calculated limit.

This method also calculates the Mahalanobis distance as a measure to identify outliers. In the studied dataset, none of the points were found to exceed the threshold, as shown in Figure 8.

After calculating the principal components, MLR can be applied using the new variables. Eight out of the twelve principal components were used since they captured 99.3% of original data’s explainability (see Table 2).

The results of MLR using eight principal components are presented in Table 3. A Box–Cox transformation of the response was performed since the residuals did not exhibit homoscedasticity, as observed in the plot of residuals vs. fitted values, which showed increasing variance instead of constant variance. It is important to note that the VIF values are low, the equation has stable coefficients, and R^2^ does not decrease from regular to prediction and 10-fold validation calculations.

Regression Equation using Principal Components

$$\frac{-1}{\sqrt[{2}]{Power_{consumption}}}=-0.094960+0.004489\;PC1+0.000221\;PC2+0.002049\;PC4\;+0.000870\;PC5-0.000822\;PC1\times PC4-0.001372\;PC2\times PC5\;$$

Regression Equation using original variables

$$\frac{-1}{\sqrt[{2}]{{Power}_{consumption}}}=-94.96+-94.96-1.18\cdot Lactose\_moisture-1.17\cdot API\_d50-\;1.34\cdot Time\_premix+3.10\cdot Time\_kneading$$

Second-order terms are excluded in the equation using the original variables for simplification.

**Figure 9:**
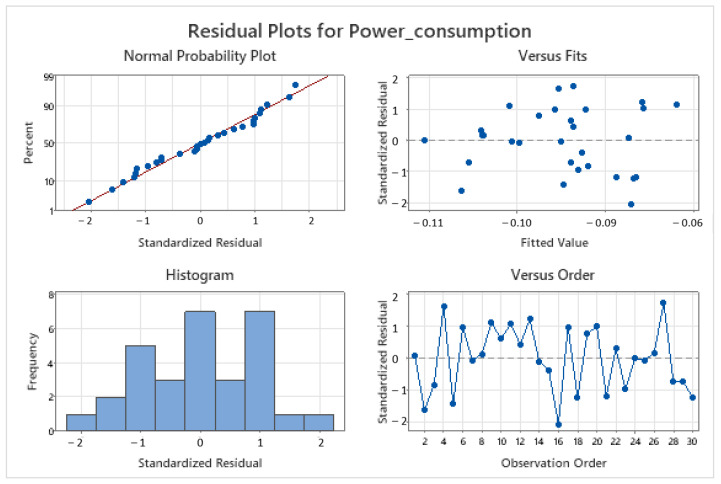
Linear model (least squares family) requirement validation.

This model meets the requisites of linear models: linearity, normality, homoscedasticity, and independence. The graphical validation is displayed in Figure 9.

### 5.3. LASSO Regression

This method enhances predictor selection with a level of objectivity that risk analysis studies may not offer. This is a clear advantage that other statistical methods do not provide. This method should be employed when multicollinearity exists to reduce the overestimation of the standard error.

**Figure 10:**
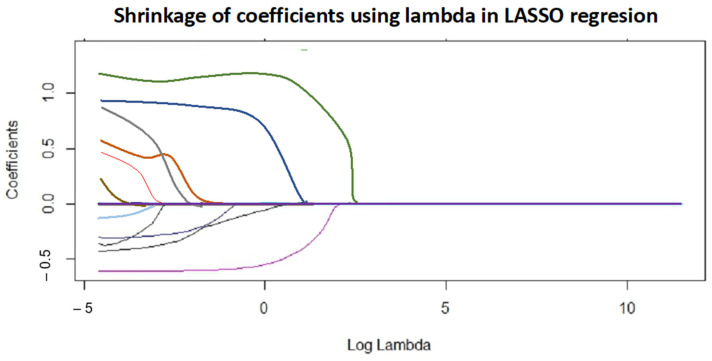
Model coefficient shrinkage with the change in lambda. Predictors kneading time (green line) and lactose_d50 (magenta line). Other lines represent the other variables (CMAs and CPPs).

The method’s sensitivity or aggressiveness in selecting predictors is adjusted using the lambda parameter (penalty factor). Figure 10 plots the coefficients versus log (lambda), illustrating how they shrink, potentially reaching zero.

Considering explainability, model parsimony, and variation reduction, the selected LASSO model, containing the predictors kneading time (the green line in Figure 10), lactose_d50 (the magenta line in Figure 10), and its second-term parameters (quadratic terms and interaction between the two main parameters), exhibits good properties for both process understanding and predictability.

The effect of kneading time is obvious and has also been highlighted by previous statistical models. When it comes to lactose_d50, it describes the mean particle size of the principal excipient, which is directly linked to its ability to adsorb a fixed amount of binding solution.

Finally, the R^2^ of the LASSO regression model is not as high as that obtained using the previous methods, but the equation has more stable coefficients and is well-suited for prediction purposes, such as anticipating process deviations and optimizing the process.

The results for the three models studied appear in Table 4, where it can be seen that if the MLR method were used to predict the quality of future batches, it would not be the best option and could result in prediction errors.

A summary of MLR, PC-MLR, and LASSO is given in Table 4.

## 6. Conclusions

The MLR method is weaker in the face of influential values, and multicollinearity has a stronger impact, resulting in statistical models with lower accuracy and limited predictability. Design spaces calculated using this method with retrospective data could be inappropriate. The deletion of influential values helps improve the accuracy and predictability of MLR-based models. On the other hand, avoiding variables with VIF > 5 is a conservative way to obtain more stable models, but often, the resulting outcome could exhibit excessive parsimony, or it may even be the case that no model can be obtained.

When MLR is used with principal components, collinearity disappears. PCA additionally provides more information about CMAs, CPPs, and CQAs, showing how they are related, thus enhancing process understanding.

Finally, LASSO regression adds objectivity, as this method allows for the distinguishing of relevant CMAs and CPPs from those with a low impact on the responses. This feature provides an additional piece of relevant information and becomes useful for introducing objectivity into previous expertise-based scenarios. Coefficients are more stable, and equations show greater explainability (measured as R^2^) and predictability (using prediction-R^2^ from cross-validation).

Although not included in this article, one more regression model is worth considering: ridge regression, also known as L2 regularization. This model reduces the coefficients showing collinearity, similar to LASSO, but the shrinkage it causes does not reach zero. Ridge regression compensates for collinearity but keeps all coefficients in the equation, not aiding in the objective selection of CMAs and CPPs with true relevance to the CQAs.

The aim of this study is to explore ways to calculate process space design and create awareness about common pitfalls that are not always well known and understood. As the statistician George Box once said, “*All models are wrong, some are useful*”. The combined use of the techniques explored in this article allows us to approach the mathematical modeling of nature using retrospective data instead of simply discarding and starting over with prospective experimentation.

## Figures and Tables

**Table 1:** Values of VIF for each parameter, and in the last part, the regular, prediction, and 10-fold R^2^.

Term	Coef	SE Coef	T-Value	*p*-Value	VIF
Constant	541	242	2.23	0.037	
Lactose_d10	−0.539	0.218	−2.47	0.022	1.15
Time_kneading	21.399	0.495	43.22	0.000	1.17
Lactose_moisture	−220.6	82.2	−2.69	0.014	7762.22
Lactose_d50	−6.05	2.41	−2.51	0.020	56.97
Lactose_moisture × Lactose_moisture	4.68	1.80	2.59	0.017	121.46
Lactose_moisture × Lactose_d50	1.978	0.815	2.43	0.024	7593.49
	S	R-sq	R-sq (adj)	R-sq (pred)	10-Fold R-sq
	1.88260	99.03%	98.75%	78.27%	78.24%

**Table 2:** To decode the mathematical model given, PCs can be substituted for the linear combinations of original variables according to this table.

Variable	PC1	PC2	PC3	PC4	PC5	PC6	PC7	PC8
Lactose_d10	0.063	0.596	0.063	−0.184	−0.033	0.253	−0.266	0.015
Lactose_d50	0.297	−0.272	−0.159	−0.140	−0.387	−0.387	−0.071	−0.439
Lactose_d90	−0.107	−0.460	−0.459	0.120	0.135	0.110	0.022	0.286
Lactose_moisture	0.036	−0.174	0.341	−0.484	−0.361	0.462	0.052	−0.014
API_d10	−0.219	−0.162	0.544	0.193	−0.121	−0.256	0.237	−0.293
API_d50	−0.275	−0.325	0.130	0.244	−0.413	0.367	−0.121	0.267
API_d90	−0.380	0.181	0.266	0.402	0.142	−0.056	0.036	−0.018
Impeller_speed_premix	0.001	0.342	−0.229	0.039	−0.465	−0.120	0.714	0.294
Time_premix	−0.304	0.003	−0.302	−0.071	0.199	0.450	0.375	−0.629
Impeller_speed_kneading	0.079	−0.211	0.298	−0.436	0.475	−0.101	0.392	0.283
Time_kneading	0.512	−0.023	0.120	0.368	0.064	0.250	0.128	−0.035
Power_consumption	0.516	−0.051	0.118	0.329	0.097	0.261	0.170	−0.064

**Table 3:** Coefficients for Transformed Response, *p*-values, VIF, and R^2^ results for the model PCR. Equation of the model.

Term	Coef	SE Coef	T-Value	*p*-Value	VIF
Constant	−0.094960	0.000328	−289.94	0.000	
PC1	0.004489	0.000242	18.53	0.000	1.45
PC2	0.000221	0.000273	0.81	0.427	1.44
PC4	0.002049	0.000294	6.96	0.000	1.02
PC5	0.000870	0.000310	2.81	0.010	1.00
PC1 × PC4	−0.000822	0.000251	−3.28	0.003	1.73
PC2 × PC5	−0.001372	0.000273	−5.02	0.000	1.45
Model Summary for Transformed Response					
	S	R-sq	R-sq (adj)	R-sq (pred)	10-Fold R-sq
	0.0017939	98.30%	98.08%	96.84%	96.78%

**Table 4:** Comparative table of R^2^ for the 3 studied models.

Model	R-sq	R-sq (adj)	R-sq (pred)	10-Fold R-sq
MLR	99.03%	98.75%	78.27%	78.24%
MLR on PC	98.30%	98.08%	96.84%	96.78%
LASSO	97.08%	96.81%	96.79%	96.79%

## Data Availability

Data are unavailable due to privacy restrictions.

## Supplementary Materials

The following supporting information can be downloaded at: https://www.mdpi.com/article/10.3390/pharmaceutics15112629/s1, File S1: Python code for used algorithms.
