# Supplementary material for: Methods for Developing a Process Design Space Using Retrospective Data
Source: Pharmaceutics. 2023 Nov 16;15(11):2629. doi: 10.3390/pharmaceutics15112629 (PMC10675834; doi:10.3390/pharmaceutics15112629)
Supplement: Supplementary file 1 [file pharmaceutics-15-02629-s001.zip › pharmaceutics-2681233-supplementary.pdf]

File S1: Python code for used algorithms

LASSO regression script:

```
from sklearn.linear_model import Lasso
from sklearn.model_selection import train_test_split
from sklearn.metrics import mean_squared_error
import numpy as np

# Load the dataset
X, y = load_dataset()

# Split the dataset into training and testing sets
X_train, X_test, y_train, y_test = train_test_split(X, y, test_size=0.2,
random_state=42)

# Create a Lasso regression model
lasso = Lasso(alpha=0.1)

# Fit the model to the training data
lasso.fit(X_train, y_train)

# Make predictions on the testing data
y_pred = lasso.predict(X_test)

# Calculate the mean squared error
mse = mean_squared_error(y_test, y_pred)
print("Mean Squared Error:", mse)

# Get the coefficients of the Lasso regression model
coefficients = lasso.coef_
print("Coefficients:", coefficients)
```

Comments:

The scripts uses the libraries **sklearn.linear_model**, **sklearn.model_selection**, **sklearn.metrics** and **numpy**.

The dataset is loaded and split it into training and testing sets using **train_test_split()**. The LASSO regression model with an alpha value of 0.1 (see next script for a variable lambda setting to select optimal factors reduction).

The model is trained using the function **fit()**. Afterwards predictions are made on the testing data using **predict()**. The mean squared error is calculated using **mean_squared_error()**. Finally, the mean squared error and the coeficients of the LASSO regression are displayed.

False Discovery Proportion vs lambda parameter in LASSO regression script:

```
from sklearn.linear_model import Lasso
from sklearn.model_selection import train_test_split
from sklearn.metrics import mean_squared_error
import numpy as np

# Load the dataset
X, y = load_dataset()

# Split the dataset into training and testing sets
X_train, X_test, y_train, y_test = train_test_split(X, y, test_size=0.2,
random_state=42)

# Define a list of lambda values to try
lambda_values = [0.01, 0.1, 1, 10]

# Iterate over lambda values
for lambda_val in lambda_values:
    # Create a Lasso regression model with the current lambda value
    lasso = Lasso(alpha=lambda_val)

    # Fit the model to the training data
    lasso.fit(X_train, y_train)

    # Make predictions on the testing data
    y_pred = lasso.predict(X_test)

    # Calculate the mean squared error
    mse = mean_squared_error(y_test, y_pred)
    print("Lambda:", lambda_val)
    print("Mean Squared Error:", mse)

    # Get the coefficients of the Lasso regression model
    coefficients = lasso.coef_
    print("Coefficients:", coefficients)
```

Comments:

This script is a modification of the previous one where a list of alfa values is used to see its effect on the False Discovery Proportion (FDP) provided by each LASSO regression solution.

This modified script shows the mean squared error and the coefficients for each alfa so that different alfa values affect the model's performance and the sparsity of the coefficients. The FDP refers to the proportion of falsely selected variables, and it can be indirectly assessed by examining the coefficients and their magnitudes.

LASSO regression with cross-validation to select optimal regularization factor:

```
from sklearn.linear_model import LassoCV
from sklearn.model_selection import train_test_split
from sklearn.metrics import mean_squared_error
import numpy as np
import matplotlib.pyplot as plt

# Load the dataset
X, y = load_dataset()

# Split the dataset into training and testing sets
X_train, X_test, y_train, y_test = train_test_split(X, y, test_size=0.2,
random_state=42)

# Create a LassoCV regression model
lasso = LassoCV(cv=5)

# Fit the model to the training data
lasso.fit(X_train, y_train)

# Get the lambda (alpha) values used during cross-validation
lambdas = lasso.alphas_

# Get the mean squared error for each lambda value
mse_values = lasso.mse_path_.mean(axis=1)

# Plot the sum of squared error vs lambda
plt.plot(lambdas, mse_values)
plt.xlabel('Lambda (alpha)')
plt.ylabel('Mean Squared Error')
plt.title('Sum of Squared Error vs Lambda')
plt.show()

# Get the coefficients of the Lasso regression model for the best lambda
best_lambda = lasso.alpha_
best_model = Lasso(alpha=best_lambda)
best_model.fit(X_train, y_train)
coefficients = best_model.coef_

# Plot the coefficients vs lambda
plt.plot(lambdas, coefficients)
plt.xlabel('Lambda (alpha)')
plt.ylabel('Coefficients')
plt.title('Coefficients vs Lambda')
plt.show()
```

Comments:

In this updated code the **LassoCV** class is used instead of **Lasso** to perform the LASSO regression with cross-validation. This script includes a 5-fold cross-validation and automatically selects the best lambda value based according to the outcome of the cross-validation.

After fitting the model to the training data, the lambda values used during cross-validation (**lasso.alphas_**) and the mean squared error for each lambda value

(**lasso.mse_path_.mean(axis=1)**) are retrieved and kept. A plot showing the sum of squared error vs lambda is displayed using **matplotlib.pyplot.plot()**.
Next, the best LASSO regression model with the best lambda value (**best_lambda**) is fit and trained. Coefficients of this best model (**best_model.coef_**) are plotted vs lambda.

This modification of hte previous algorithm allows to see how the sum of squared error and the coefficients change with different lambda values, providing insights into the trade-off between model complexity and error.
